# Feline Susceptibility to Leptospirosis and Presence of Immunosuppressive Co-Morbidities: First European Report of *L. interrogans* Serogroup Australis Sequence Type 24 in a Cat and Survey of *Leptospira* Exposure in Outdoor Cats

**DOI:** 10.3390/tropicalmed8010054

**Published:** 2023-01-10

**Authors:** Elisa Mazzotta, Gabrita De Zan, Monia Cocchi, Maria Beatrice Boniotti, Cristina Bertasio, Tommaso Furlanello, Laura Lucchese, Letizia Ceglie, Laura Bellinati, Alda Natale

**Affiliations:** 1 Istituto Zooprofilattico Sperimentale delle Venezie (IZSVe), Viale dell’Università 10, 35020 Legnaro, Italy; 2 National Reference Centre for Animal Leptospirosis (NRCL), Istituto Zooprofilattico Sperimentale della Lombardia ed Emilia Romagna “Bruno Ubertini”, Via Bianchi 7/9, 25121 Brescia, Italy; 3 Veterinary Clinic and Laboratory San Marco, Viale dell’Industria, 35030 Veggiano, Italy

**Keywords:** *Leptospira*, cat, co-morbidities, immunosuppression, susceptibility, microscopic agglutination test (MAT), real-time PCR, multi locus sequence typing

## Abstract

Leptospirosis is one of the most widespread zoonotic diseases and can infect both humans and animals worldwide. The role of the cat as a susceptible host and potential environmental reservoir of *Leptospira* is still not well understood, due to the lack of obvious clinical signs associated with *Leptospira* spp. infection in this species. This study aims to describe the first European detection of *Leptospira interrogans* serogroup Australis ST 24 in a young outdoor cat with a severe comorbidity (feline panleukopenia virus). In addition, the results of a preliminary study conducted in 2014–2016 are presented (RC IZSVE 16/12), which reports an investigation of *Leptospira* exposure of outdoor cats in Northeast Italy by means of serological investigation and molecular evaluation of urine. The animals included in the survey are part of samples collected during active and passive surveillance (diagnostic samples). The study reported a seroprevalence of 10.5% among outdoor cats and the serogroups identified were Grippotyphosa, Icterohaemorrhagiae, Bratislava, Canicola and Ballum. Symptomatic cats reported high MAT titres (ranging from 1:800 to 1:1600) towards antigens belonging to the serovars Grippotyphosa (1:800), Bratislava (1:1600), Icterohaemorrhagiae (1:200) and Copenhageni (1:200–1:800). In one subject, urine tested positive for *Leptospira* PCR. Cats with high antibody titres for *Leptospira* and/or positivity on molecular test suffered from immunosuppressive comorbidities (feline immunodeficiency virus and feline leukaemia virus; feline herpesvirus and lymphoma; hyperthyroidism). The overall prevalence of serum antibodies against *Leptospira* found in free-ranging cats (10.53%, 95% CI: 4.35–16.70%) and the identification of *L. interrogans* ST 24 in a young cat with immunosuppressive disease (feline panleukopenia virus) suggest the possibility of natural resistance to clinical leptospirosis in healthy cats. In a One Health perspective, further studies are needed to better define the pathogenesis of leptospirosis in cats and their epidemiological role as environmental sentinels or possible carriers of pathogenic *Leptospira*.

## 1. Introduction

Leptospirosis is caused by spirochaetal bacteria of the genus *Leptospira* and is an almost endemic disease worldwide. *Leptospira* spp. potentially affects all mammals that can act either as primary/defective hosts, which develop the acute disease, or as carrier hosts, which are primarily responsible for spreading the disease. The genus *Leptospira* is currently divided into three phylogenetic clusters, which supposedly correlate with the virulence of the bacteria [1,2]. To date, at least 64 *Leptospira* species have been described, and these have been classified into four subclades (S1, S2, S3 and S4), including more than 300 serovars [3,4,5]. Recently, 43 novel species of *Leptospira* were isolated from tropical soils, suggesting a highly unexplored biodiversity in the genus [4,6,7]. Leptospirosis occurrence is related to specific climatic conditions (i.e., moist soils with neutral pH and warm temperature around 25 °C) that could allow leptospires to persist and remain infectious for several months, leading to potentially relevant environmental contamination [1,4,8,9].

The serogroups Australis, Autumnalis, Ballum, Canicola, Grippotyphosa, Icterohaemorrhagiae, Pomona and Sejroe are the most frequently reported among cats in Europe, although with significant regional variation, both in terms of serovars and hosts distribution [9,10]. The overall prevalence reported worldwide by microscopic agglutination tests (MAT) ranges from 4% to 33.3%, whereas molecular assays have reported widely different prevalence values, probably influenced by the molecular techniques employed (i.e., PCR-primers) and the characteristics of the geographical area [9]. Antibodies against *Leptospira* spp. are mostly reported in older, outdoor, urban cats with hunting behaviour [9,11,12,13]. In addition, a recent serologic and urinary survey in Canada reported that exposure to *Leptospira* was unexpectedly relevant in feral cats [14]. 

Cats are now considered to represent a possible zoonotic risk, although the clinical course of the disease related to the pathogenesis of leptospirosis in this species is still not fully elucidated [8,9,10]. Previous literature reported that the cat could be a potential chronic reservoir host, since, in urine, *Leptospira* can be isolated and its DNA can be identified for more than 8 months, even when the infected cat presents detectable specific serum antibodies [15]. Concerning the cat’s natural exposure to pathogenic *Leptospira*, the most recent literature reported that in Europe, the serological prevalence of anti-*Leptospira* antibodies varies geographically: 14% in Andalusia [16], 12.8% in Estonia [17], 4.1% in Spain [18] and 9.2% in the Czech Republic [19]. Previous studies reported a *Leptospira* serological positivity of 18.2% in Austria (Tyrol’s districts) [20] and 9.2% in Scotland [11]. Recently, a seroprevalence of 17.9% was described in Germany in outdoor cats, with titres ranging from 1:100 to 1:6400. The most common serovars were Australis (8.7%, MAT titres range 1:100–1:6400), Bratislava (7.2%, range 1:100–1:400) and Grippotyphosa (5.6%, range 1:100–1:400) [21]. 

Reports of clinical leptospirosis in cats are rare: the clinical presentation is characterised by a plethora of signs ranging from asymptomatic to fulminant disease, making the diagnostic process challenging. Specifically, the acute clinical disease mainly involves the young incidental host infected by haemolysin-producing *Leptospira* species, but in the majority of cases clinical signs are likely to be mild [10,22]. Experimentally described clinical signs include polyuria, polydipsia, haematuria, uveitis, lethargy, anorexia, weight loss, ascites, vomiting, diarrhoea, pain on handling, inflammatory lesions on the skin and digits, cavity effusions, and nephritis, although infection in cats is not always associated with suggestive symptoms [10,23,24]. Clinicopathologic changes are a fluctuating leukocytes count, azotaemia, hypokalaemia, hyperphosphatemia, hyposthenuria, haematuria and proteinuria. Therefore, while the interstitial nephritis appears a common clinical manifestation, the liver dysfunction is not reported as frequently as in dogs [9,24,25].

Outdoor behaviour and predatory activity seem to be the most relevant risk factors. In addition, living in rural areas in close contact with livestock farms, contact with synanthropic or wildlife species and the presence of other cats in the household are also associated with an increased risk of leptospirosis [9,12]. Further environmental risk factors have been identified, such as living in flooded areas where agricultural activities use water flowing in streams or backwaters [26]. Accordingly, in tropical countries, the frequency of clinical manifestations of leptospirosis in people is strongly correlated with rainy seasons: flooding contributes strongly to disease transmission, since a high degree of flooding leads to more infected individuals [27,28]. 

The presence of diseases associated with immunosuppression or immune system deficiency can lead to decreased resistance to infection, debilitation and other complications of illness. Varieties of infectious and non-infectious diseases, stress, poor nutrition, drugs, toxins and medical procedures have been associated with immunosuppression in dogs and cats [29]. Specifically, the viral agents that could commonly affect outdoor and shelter cats are feline panleukopenia virus (FPV), feline herpesvirus, feline caliciviruses (FCV), feline infectious peritonitis virus (FIPV), feline leukaemia virus (FeLV) and feline immunodeficiency virus (FIV) [30]. FIP is caused by a feline coronavirus (FCoV) in which the immune system is known to play a crucial, but complex, role in the pathogenesis. This role is still not fully understood, with involvement of both host and viral factors [31]. Young cats of less than 2 years of age seem especially vulnerable, and it has been estimated that 0.3% to 1.4% of feline deaths at veterinary institutions are caused by FIP [32]. FeLV and FIV infections are reported in cats worldwide. Both infections are associated with a variety of clinical signs and can impact quality of life and affect longevity [33]. The major route of FIV transmission is through bite wounds that introduce saliva containing virus and FIV-infected white blood cells. Following the primary phase of the infection, cats enter a long asymptomatic stage that can last for many years. During this chronic stage, progressive dysfunction of the immune system can occur. Thus, FIV-infected cats are predisposed to chronic and recurrent infections. Moreover, neoplasia is about five times more common than in uninfected cats [34]. Infection with FeLV is transmitted through close contact among cats. Commonly, it is spread vertically and horizontally from infected queens to their kittens and horizontally among cats that live together or that fight [34]. The prevalence in individually kept cats is usually less than 1%; differently, in multi-cat households, this figure may be 20%: the high density of cat populations represents a facilitating factor [35]. Immune suppression in FeLV infections is more complex and severe than the more selective effects caused by FIV. Whether showing clinical signs or not, every FeLV-viraemic cat is immune suppressed, with retarded and decreased primary and secondary antibody responses [36]. FPV may affect cats of all ages, but kittens are most susceptible. FPV has become less frequent in the domestic cat population over the last decades because of vaccination. However, outbreaks in shelter cats are commonly reported and often associated with a high number of fatalities [37]. Signs of disease include diarrhoea, lymphopenia and neutropenia, followed by thrombocytopenia and anaemia, immunosuppression (transient in adult cats), cerebellar ataxia (in kittens only) and abortion [38]. Feline panleukopenia mortality is 25–90% in cats with the acute form of the disease and up to 100% in hyperacute infections [39].

To the best of the author’s knowledge, there are no recent studies about the possible association between immunosuppressive infectious disease and clinical leptospirosis. A proportion of the cat population in Italy has outdoor access, where they have the opportunity to hunt prey [40]. Therefore, a large population of outdoor cats could be exposed to *Leptospira* spp. and might play a role in the complex epidemiology of the disease. Only a few recent studies have described the most commonly circulating strains of *Leptospira* spp. among dogs in Northeast Italy [41], whereas no recent reports of serological investigation are known in cats in these regions. 

The present study reports the first identification of *L. interrogans* serogroup Australis ST 24 in an immunocompromised young cat and describes four suspected clinical cases of feline leptospirosis. Furthermore, the results of a preliminary serological and epidemiological survey of *Leptospira* in free-roaming cats in Northeast Italy are described.

## 2. Materials and Methods

### 2.1. Diagnostic in a Young Cat Referred for Sudden Death

A 4-month-old cat was referred from the veterinarian practitioner to the Istituto Zooprofilattico Sperimentale delle Venezie (IZSVE) laboratory for post-mortem examination. The cat was a young free-roaming male domestic short hair from a shelter. The cat had been receiving itraconazole (Itrafungol^®^, Virbac, Milan, Italy) for dermatomycosis, which was previously diagnosed by the veterinary surgeon. The cat presented severe and acute gastrointestinal syndrome with vomiting, haemorrhagic diarrhoea, anorexia and dehydration, which rapidly deteriorated, leading to his death within hours. 

#### 2.1.1. Microbiological Analysis

Based on the necropsy findings, selected organs were sampled for further diagnostic investigations. Specifically, swabs from faeces, liver and lung were submitted to laboratory routine aerobic and anaerobic culture; faeces and a pool of visceral organs (liver, gall bladder and spleen) were analysed for *Salmonella* spp. isolation, according to the World Organization for Animal Health (WOAH), Chapter 3.10.07 [42]. Bacterial identification was phenotypically performed using a routine test. 

*Leptospira* isolation was attempted on liver, kidney and lung tissue. A sample of 1 cm^3^ of these organs was homogenized with a pestle and mortar and added to 9 mL of liquid Ellinghausen–McCullough–Johnson–Harris (EMJH) medium, according to WOAH, Chapter 3.1.12 [3]. 

#### 2.1.2. Molecular Analysis and Genome Sequencing

The liver, lung and kidney tissue samples were assessed via a pathogen-specific *Leptospira* TaqMan real-time polymerase chain reaction (real-time PCR) kit. The tissue samples were homogenized at a 1:10 dilution in 600 µL of PBS, with TissueLyser II (QIAGEN, Hilden, Germany), and DNA isolation from 100 µL of tissue homogenate was performed after a prelysis treatment with 2.5 μL of lysozyme (10 mg/mL in 10 mM of Tris-HCl, pH 8.0) and an incubation period of 15 min at +37 °C. The DNA extraction was performed on the KingFisher™ Flex Purification System (Life Technologies, Carlsbad, CA, USA) platform using the ID Gene^®^ Mag Universal Extraction Kit (IDvet, Grabels, France), in accordance with the manufacturer’s instructions. Every DNA extraction included a negative process or extraction control (water). To detect the presence of pathogenic species of *Leptospira*, a screening real-time PCR targeting a 87 bp fragment that corresponds to a portion of the gene encoding the 16S rDNA was applied [43]. The real-time PCR was performed in a 25 µL final volume, containing 3 µL of extracted DNA, 12.5 µL of 2× Master Mix TaqMan Universal 2× (Thermo Fisher Scientific, Waltham, MA, USA), 300 nM of each primer, and 100 nM of a 5′ 6-carboxyfluorescein (FAM)–3′-tetramethylrhodamine (TAMRA) probe. The amplification assay included a negative control (water), a negative bacterial genomic control (DNA of *Leptospira* biflexa sv Patoc), the negative extraction control and a positive control (DNA of *L. interrogans* sv Icterohaemorrhagiae). The assay was performed with the following thermal conditions: a holding step at 95 °C for 10 min, and 45 cycles of 95 °C for 15 s and 60 °C for 60 s. Samples with cycle threshold (Ct) < 38 were considered positive. Samples with Ct values within the 38–40 range were considered doubtful, whereas samples with no FAM fluorescence signal or with Ct ≥ 40 were considered negative.

The positive samples were referred to the Istituto Zooprofilattico Sperimentale della Lombardia e dell’Emilia Romagna (IZSLER) National Reference centre for Leptospirosis for the genomic characterization with the multi locus sequence typing (MLST) technique. The genotyping was performed using the 7-loci scheme proposed by Boonsilp in 2013 [44], which is based on the housekeeping genes glmU, pntA, sucA, tpiA, pfkB, mreA and caiB, as previously described [41]. 

For allelic number and ST identification, the assembled and trimmed sequences were queried against the Bacterial Isolate Genome Sequence Database (BIGSdb) available on the Leptospira MLST website (https://pubmlst.org/leptospira/, accessed on 1 March 2022), sited at the University of Oxford [45]. Comparisons between the STs found and those present in BIGSdb as reference isolates were used to deduce the species of the Leptospira being tested. To perform comparisons among historical serological studies (where serovars and serogroups were defined) and genotyping data (where species and genomic profiles were defined), we chose to assign to each identified ST a classification at the serogroup and serovar levels obtained from BIGSdb, knowing that this information was deduced and did not result from active serological typing.

A pool of tissue samples from the stomach and small intestine were submitted to quantitative molecular testing for FPV [46]. The tissue samples were homogenized at a 1:10 dilution in 800 µL of PBS supplemented with antibiotics (PBS-A: 10,000 IU/mL of penicillin G, 10 mg/mL of streptomycin, 5000 IU/mL of nystatin and 0.25 mg/mL of gentamicin sulphate), with TissueLyser II (QIAGEN, Hilden, Germany) at 30 Hz for 3 min. The DNA extraction was performed on the KingFisher™ Flex Purification System (Life Technologies, Carlsbad, CA, USA) platform using the ID Gene^®^ Mag Universal Extraction Kit (IDvet, Grabels, France), in accordance with the manufacturer’s instructions, adding a pre-treatment with 20 µL of Proteinase K(QIAGEN, Hilden, Germany) for 10 min at 70 °C before the extraction. Every DNA extraction included a negative control (water). A real-time PCR on a conserved region of gene VP2 of FPV [46] was carried out in a final volume of 25 µL, consisting of 5 µL of eluted DNA, 5 µL of Quantifast Pathogen Master Mix 5× (QIAGEN, Hilden, Germany), 2.5 µL of Internal Control Assay 10× (QIAGEN, Hilden, Germany), 600 nM of each primer and 200 nM of probe. The assay was performed with the following thermal conditions: a holding step at 95 °C for 5 min, followed by 40 cycles at 95 °C for 15 s and 59 °C for 30 s. The real-time PCR was performed on a Biorad CFX96 instrument (Biorad, Hercules, CA, USA). Samples with cycle threshold (Ct) < 35 were considered positive, whereas samples with no FAM fluorescence signal or with Ct ≥ 35 were considered negative.

### 2.2. Ricerca Corrente IZSVE 16/12: Sample Collection from 2014 to 2016

The study design involved the collection of samples according to both active and passive epidemiological survey. Ninety-five samples were collected from free-roaming cats with no suspected *Leptospira* infection reported (active surveillance), while four cases were reported as cases of suspected leptospirosis (passive surveillance). *Leptospira* investigation of the free-roaming cats (*n* = 95) was conducted by retrospectively testing the cat samples collected for other purposes (health status control, pre-surgery investigation, other diagnostics investigations) and enrolled for the study. The test included *Leptospira* antibody detection by means of a microscopic agglutination test (MAT) on serum [3] and a real-time PCR on urine for the detection of pathogenic *Leptospira*, as previously described. The samples were collected from 2014 to 2016 by the veterinary service of the health authority in Northeast Italy (Veneto region) and by veterinary private practices: the samples were submitted to the laboratory of IZSVE and tested for *Leptospira* through serological and/or molecular methods, as reported above. Anamnestic data, including the clinical features, of the cats enrolled for the study were collected when available. The average age of these subjects was 5.95 years (standard deviation ± 3.75), accounting for 34 young cats (age from 1 to 3 y/o), 42 adult cats (age from 4 to 8 y/o) and 19 senior cats (age > 9 y/o). The study population enrolled 47 female cat (47/95, 49.67%, 95% CI: 36.42–59.53%), and 48 male cat (48/95, 50.53%, 95% CI: 40.47–60.58%).

Moreover, as part of the passive surveillance, samples from suspected clinical cases came from veterinary clinical practices. The clinical symptoms included polyuria, polydipsia, kidney disease and dehydration. The samples (EDTA-whole blood, serum and urine) were collected during routinely diagnostic procedures carried out by the veterinary surgeons. In this context, considering the specific case, it was not deemed necessary to submit a specific request to the Ethics Committee. All procedures complied with the ethical standards of the relevant national and European regulations on animal welfare. Data concerning the cat’s clinical features and the presence of co-morbidities were reported by the attending veterinarians. 

#### Serological Analysis: Micro Agglutination Test (MAT)

All the collected serum samples (*n* = 99, active and passive surveillance) were submitted to MAT, according to the WOAH method (Chap 3.1.12) [3]. The antigen panel included 8 serogroups and 9 serovars distributed by the Italian Reference Centre for Animal Leptospirosis as antigens in the routine diagnostic MAT (*L*. *interrogans* serogroup (sg) Australis serovar Bratislava; *L*. *interrogans* sg Canicola serovar Canicola; *L*. *kirschneri* sg Grippotyphosa serovar Grippotyphosa; *L*. *interrogans* sg Icterohaemorrhagiae serovar Copenhageni; *L*. *interrogans* sg Icterohaemorrhagiae serovar Icterohaemorrhagiae; *L*. *interrogans* sg Pomona serovar Pomona; *L*. *interrogans* sg Sejroe serovar Hardjo; *L*. *borgpetersenii* sg Tarassovi serovar Tarassovi; *L*. *borgpetersenii* sg Ballum serovar Ballum) [47].

The serum samples were pretested at the final dilution of 1:100. Samples with 50% agglutination were retested to determine an endpoint using dilutions of serum beginning at 1:100 through to 1:6400. Serum samples with the widely accepted minimum significant titre of 1:100 (reciprocal of the final dilution of serum with 50% agglutination) were assessed as positive. In cases of clinically suspected *Leptospira* infection, urine samples were collected and analysed for bacterial culture and *Leptospira* isolation (*n* = 2). In addition, these urine samples were assessed via a pathogen-specific *Leptospira* TaqMan (real-time PCR) kit [43], as previously described [41]. 

## 3. Results

### 3.1. Leptospira Interrogans Serogroup Australis ST 24 in an Immunocompromised Cat

**Figure 1:**
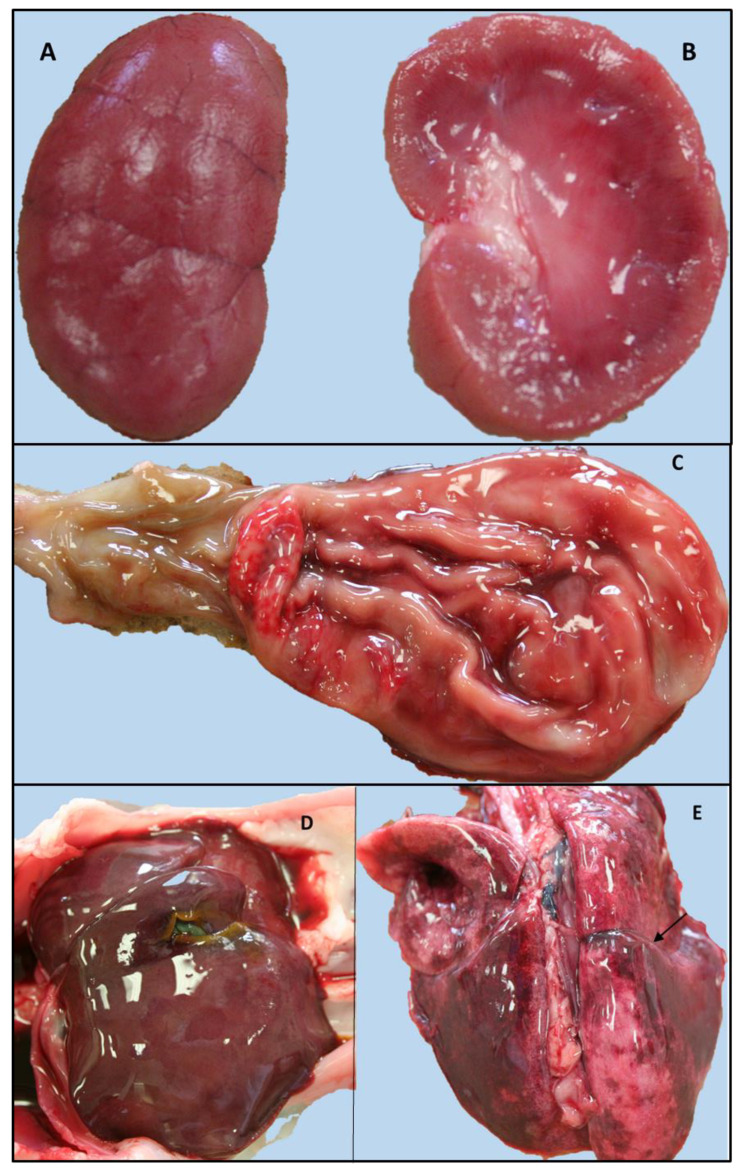
Gross findings of the kitten positive for FPV and *Leptospira interrogans* serogroup Australis ST 24. Left kidney dorsal and longitudinal section: multifocal, ill-defined cortico-midline demarcation and a prominent renal cortex with multifocal, rarely coalescent, bright red radial striations (**A**,**B**). (**B**) Stomach and proximal intestine (duodenum): catarrhal-haemorrhagic gastritis, erosive alteration of the mucosa of the pyloric region. (**C**) Increased size of the liver with blurred margins and slight diffuse prominence of the parenchyma. (**D**) Severe pulmonary edema and haemorrhagic suffusions: area of adhesion between the middle and caudal lobes of the right lung (arrow) (**E**).

The gross pathology main findings included moderate, acute, catarrhal-haemorrhagic gastritis with areas of erosion in the pyloric region, as well as severe segmental catarrhal-haemorrhagic enteritis of the proximal enteric tracts (duodenum and digiunum). Moderate hepatomegaly was observed in association with mild and diffuse prominence of the parenchyma, as well as multifocal and irregular areas of greyish discoloration with ill-defined margins. The kidney showed multifocal areas of poorly defined cortico-medullary demarcation. The renal cortex was prominent with multifocal, rarely coalescing, bright red radial striations. A moderate quantity of sero-haemorrhagic pleural effusion was reported in both hemithorax, and the lungs were bilaterally characterized by multifocal to locally diffuse, dark-red, irregular and haemorrhagic areas associated with pulmonary edema. In addition, an extensive area of adhesion between the middle and caudal lobes of the right lung was described, associated with pleural fibrin and a moderate increase in parenchymal consistency (Figure 1). Finally, the mucous membranes appeared pale and the peripheral lymph nodes and tonsils were moderately increased in volume with prominent follicles on the cut surface. Unfortunately, histopathology was not performed, as reported in the study limitation paragraph. 

Bacteriological samples from intestine, liver and lung were positive for Escherichia coli, whereas Salmonella spp. analysis tested negative. Finally, gastrointestinal samples tested positive for the molecular detection of FPV (Ct 23.01) (stomach and small intestine tissue samples). 

Bacterial culture tested negative for *Leptospira* spp. (kidney, lung and liver).

The molecular analysis reported positivity for *Leptospira* spp. only in the kidney tissues (Ct 35.95), while lung and liver resulted negative. The *Leptospira* DNA, submitted to an MLST analysis, was typed as *Leptospira* ST 24, which identifies *L*. *interrogans* serogroup Australis. 

### 3.2. Survey on the Exposure to Leptospira spp. of Feline Populations in Northeast Italy (2014–2016): RC IZSVE 16/12

#### 3.2.1. *Leptospira* Investigation in Free-Roaming Cats: Active Surveillance

Ninety-five free-roaming cats were tested for anti-*Leptospira* antibodies (MAT) and for pathogenic *Leptospira* detection in urine (real-time PCR). The MAT was positive in 10 out 95 cats (10.53%, 95% CI: 4.35–16.70%), and the most representative serogroups were Grippotyphosa (*n* = 6/95; 6.32%, 95% CI: 1.42–11.21%), Icterohaemorrhagiae (*n* = 2/95; 2.11%, 95% CI: 0.00–4.99%) and Bratislava (*n* = 3/95; 3.16%, 95% CI: 0.00–6.67%). None of these cats tested positive in urine and blood by real-time PCR. The animals reported low antibody titres (<1:200), both against *L. kirschneri* sg Grippotyphosa serovar Grippotyphosa and *L. interrogans* sg Icterohaemorrhagiae serovar Icterohaemorrhagiae. One cat reported antibodies against *L. interrogans* sg Australis serovar Bratislava (titre 1:200) and one cat showed positivity for *L. interrogans* Canicola serovar Canicola (titre 1:100). One young and pregnant queen showed antibody titres against *L. kirschneri* sg Grippotyphosa serovar Grippotyphosa (titre 1:100), *L. interrogans* sg Icterohaemorrhagiae serovar Icterohaemorrhagiae (titre 1:100) and *L. interrogans* sg Icterohaemorrhagiae serovar Copenhageni (titre 1:100). Finally, a pregnant adult queen reported low titres for *L. borgpetersenii* sg Ballum serovar Ballum (titre 1:100) (*n* = 1/95, 1.05%, 95%CI 0.00–3.10) (Table 1). The real-time PCR on the urine samples tested negative.

#### 3.2.2. *Leptospira* Investigation in Free-Roaming Cats with Clinical Symptoms: Passive Surveillance

Among the four cats selected by passive surveillance showing clinical symptoms suggestive of possible *Leptospira* infection, high MAT titres (>1:400) were recorded towards serogroup/serovars Grippotyphosa (*n* = 2/5; 40%, 95% CI: 0.00–82.9%) and Bratislava (*n* = 2/5; 40%, 95% CI: 0.00–82.9%). MAT titres of 1:200 were reported for Icterohaemorrhagiae (*n* = 1/5, 20%, 95% CI 0.00–55.06%) and Copenhageni (*n* = 2/5; 40%, 95% CI: 0.00–82.9%). Moreover, two cats reported a co-presence of different serovars (*L.* Grippotyphosa, *L.* Icterohaemorrhagiae and *L.* Copenhageni; *L.* Bratislava and *L*. Copenhageni, respectively). Interestingly, the cats that were positive for *Leptospira* (high antibody titres and/or molecular positivity) presented severe immunocompromising co-morbidities, such as FIV and FeLV, FHV-1 infection and hyperthyroidism, and lymphoma. (Table 1).

## 4. Discussion

Leptospirosis in cats still has several unclear aspects. Epidemiological studies and the identification of serovars of *Leptospira* circulating in this species help to fill the gaps in the definition of the eco-pathological picture of leptospirosis and the role of the cat. Although outdoor cats are potentially easily exposed to the pathogen (predatory behaviour, contact with reservoirs and contaminated environments), they appear to be less prone to the development of clinical disease than other susceptible animals. It is not completely clarified which serovars can cause incidental infections in cats. Based on previously published reports of acute leptospirosis in cats, serovars belonging to Autumnalis, Australis, Icterohaemorrhagiae, Grippotyphosa, Pomona and Sejroe serogroups seem to be mostly involved [9]. Interestingly, during an ongoing study conducted by these authors’ research team (Mazzotta E., et al.), *L*. *interrogans* Australis ST 24 was detected in hedgehogs, mice and foxes in Northeast Italian regions, suggesting a possible prey–predator epidemiological scenario (preliminary unpublished data).

The present study describes the identification of *L. interrogans* Australis ST 24 in a kitten with severe immunosuppressive co-morbidity (FPV). The clinical symptoms (haemorrhagic diarrhoea) and the gross pathology findings (haemorrhagic enteritis) were highly suggestive of FPV infection [48,49], as confirmed by the positive real-time PCR result. FPV is transmitted via the faecal–oral route, and the infected subject is able to shed high titres of virus, rapidly contaminating the environment. Feline panleukopenia represents a severe disease, common signs of which include lymphopenia and neutropenia, followed by thrombocytopenia and anaemia, immunosuppression (transient in adult cats), neurological and reproductive symptoms [50]. In addition, this cat reported both pulmonary severe diffuse haemorrhagic lesions and hepatic alterations: bacteriological identification revealed in both lung and liver samples showed a positive high load for *E*. *coli*. The identification may indicate an extra intestinal localization of this bacterium, likely related to the poor immunity of the kitten. 

In this context, the shelter condition/household, the young age of the cat, the expected presence of immune dysfunction due to FPV and the possible presence of bacterial co/secondary infection, could likely have favoured the occurrence of *Leptospira* infection. As previously reported in the literature [9,12], this cat could be more likely considered as an incidental host rather than a chronic carrier for leptospirosis.

Concerning the epidemiological evaluation among free-roaming cats in Northeast Italy, these authors reported an apparently reassuring situation in clinically healthy cats, with sporadic seropositivity at low titres and no direct detection of *Leptospira*. Conversely, a different scenario appeared in four clinically suspected cases: these cats showed suggestive symptoms of clinical leptospirosis, high MAT antibody titres, and one animal tested positive in a real-time PCR analysis on the urine sample. All these cases presented severe systemic co-morbidities (i.e., FPV, FIV, FeLV and lymphoma). 

To the best of the authors’ knowledge, a specific correlation between *Leptospira* ST and immunosuppressive co-morbidities has not been demonstrated, but significant associations with an inflammatory condition and stress response were reported in cats exposed to *Leptospira* spp. In *Leptospira* spp. antibody-positive cats, alterations in CBC (anaemia, neutrophilia, monocytosis and eosinopenia), in inflammation markers (i.e., hypoalbuminemia and hyperglobulinemia) and increased ALT activity have been reported [51]. Previous case reports described three confirmed, naturally infected clinical cases of feline leptospirosis, in which the major clinical findings were different stages of renal insufficiency without any liver involvement [24,52]. Dissimilar information is available about the correlation between chronic kidney disease and serological positivity for *Leptospira* [10,53]. Although further investigations are needed, it is possible that the lack of an adequate immune response in animals with immunosuppressive diseases may have favoured the development of systemic leptospirosis, in association with a clinically evident condition in the most severe cases.

The free-roaming cats found positive for *Leptospira* antibodies but without any suggestive clinical symptoms reported low serological titres (>1:100): these findings would be suggestive of exposure, possibly recurrent, to pathogenic *Leptospira* or subclinical and chronic infection, since it has been previously reported that cats can demonstrate positive serology of leptospirosis months after the suspected time of infection/exposure [9]. Thus, it is possible that cats may develop clinical signs after a longer period than what has been documented experimentally [23]. Unfortunately, it was not possible to evaluate the animals afterwards, so no follow-up data are available. 

The most frequent serovars involved in feline leptospirosis in Europe, based on serological investigations and according to the European consensus statement on leptospirosis, belong to serogroups Australis, Autumnalis, Ballum, Canicola, Grippotyphosa, Icterohaemorrhagiae, Pomona and Sejroe [10]. According to this, our study showed the presence of antibodies against Grippotyphosa, Icterohaemorrhagiae, Canicola and Australis, despite these low serological titres being suggestive of exposure or subclinical infection. The comparison of the serological data available in the literature about anti-*Leptospira* antibodies in cats is likely biased by geographical origin, sampling method and the diagnostic technique applied [24,53,54]. Environmental factors, such as outdoor habits, the presence of livestock and farm animals that may shed *Leptospira* in the neighbourhood, wild animals’ *Leptospira* reservoirs, and seasonality, may result in different degrees of exposure to pathogenic *Leptospira*, thus, potentially justifying the broad ranges of antibody prevalence reported in the literature. A recent study conducted in cats in southern Italy reported antibodies against serovars Poi, Arborea and Mini, among others [51]. This study also described the spring season as the only risk factor for urinary *Leptospira* shedding, detected in 9% of urine samples. Moreover, laboratory variability determined by both the use of different methods and the application of different cut off values (≥1:100), and variations in host-specific humoral immune responses, can be a hindrance to the correct identification of positive results. Many different *Leptospira* antigens were tested in the immunoassay, but false-negative results occur when the infecting serogroups are not included. Furthermore, the significance and duration of *Leptospira* species antibodies, as detected by the MAT in cat sera, are largely unknown. It is even possible that seroconversion in cats is expressed at a lower titre compared to dogs [55]. However, the MAT is believed to be specific for *Leptospira* species antibodies, even if it is unknown whether antibodies against other spirochetes in feline sera can lead to falsely positive results. Although not completely elucidated, it has recently been experimentally assessed that antibodies produced following infection by other spirochetes in cats (i.e., *Borrelia burgdorferi*) are not detected in *Leptospira* MAT [55]. 

The evaluation of serological tests in cats is a challenge: cross-reactivity with non-vaccine serogroups has been demonstrated in dogs, as well as in cats, and antibody production after infection in cats appears to be serogroup-specific, although immune protection is not clearly understood [14]. The seroprevalence against *Leptospira* observed in our study was 10.53%, falling within the previous intervals (4% to 33.3%) described worldwide [18,26,55,56,57]. 

The cat’s role as a possible cause of *Leptospira* environmental contamination and source of exposure for people is not fully understood [9], whereas it is a susceptible host for *Leptospira* spp. and could potentially present a chronic leptospirosis infection with urinary shedding. Feline leptospirosis is likely to be underdiagnosed: it is common for leptospirosis not to be considered as a possible differential diagnosis, even in animals with clinical symptoms suggestive of acute *Leptospira* infection (i.e., acute renal failure). Moreover, the possible underestimation of leptospirosis in cats may be due to other factors, such as the challenging clinical diagnosis related to mild or atypical clinical signs, and difficulties in serological or molecular analysis. These circumstances may impact the scarcity of official reports and the perceived low prevalence of the infection within this species [58]. Therefore, the lack of a large serological survey and molecular detection among *Leptospira* spp.-positive cats does not adequately define the zoonotic risk factors nor the epidemiological role of this species.

## 5. Conclusions

The detection of new ST in cats highlights the need to consider the challenge of better understanding the effect of exposure to or infection with pathogenic *Leptospira* spp. in felines, and the need to define their epidemiological role as sentinel hosts or environmental reservoirs. Outdoor and shelter cats that are free-roaming and hunt prey would be a population worthy of special attention: although they may not explicitly manifest symptoms of leptospirosis, they may instead reveal circulating strains of *Leptospira* in a domestic–shelter (cat–prey) or domestic–domestic (cat–dog or other susceptible domestic mammals) context. To date, many questions remain to be clarified, particularly concerning the cat’s ability to be a chronic carrier rather than an environmental sentinel. In addition, as highlighted by the serological survey in outdoor cats, further studies are needed to increase knowledge about the host-immune response following infection or exposure to specific *Leptospira* serovars within the feline population. Furthermore, the evaluation of the possible aetiopathogenic link between clinical leptospirosis and immunosuppressive diseases, and the analysis of the immune pathways, would be useful to improve the diagnostic techniques.

## Figures and Tables

**Table 1:** MAT titres detected during the survey in cats (study RC 16/12); the significant clinical features and concurrent diseases (active surveillance). Clinically symptomatic cats tested positive for *Leptospira* spp. MAT and/or real-time PCR, and the presence of clinical signs and concurrent diseases (passive surveillance).

Case Log	MAT	Real-Time PCR	Bacterial Isolation	Clinical Features and Concurrent Diseases
Active surveillance				
European short hair Male neutered 6 years old	*L. kirschneri* sg Grippotyphosa serovar Grippotyphosa (titre 1:100)	Negative	Negative	Chronic renal failure Pituitary hyperadrenocorticism Hyperaldosteronism Hyperthyroidism Hypertrophic Cardiomyopathy FIV
Maine Coon Male neutered 1 year old	*L. interrogans* sg Icterohaemorrhagiae serovar Icterohaemorrhagiae (titre 1:100)	Negative	Negative	Vertebral fracture (trauma) Hepatopathy Pneumopathy
European short hair Female neutered 1 year old	*L. kirschneri* sg Grippotyphosa serovar Grippotyphosa(titre 1:100)	Negative	Negative	Acute over chronic renal failure Central neuropathy Urinary tract infection FELV FIV
European short hair Male 2 years old	*L. kirschneri* sg Grippotyphosa serovar Grippotyphosa (titre 1:100)	Negative	Negative	Bilateral otitis Dental disease
European short hair Female 5 years old	*L. kirschneri* sg *Grippotyphosa* serovar Grippotyphosa (titre 1:100)	Negative	Negative	Dental disease
European short hair Female 5 years old	*L. borgpetersenii* sg Ballum serovar Ballum (titre 1:100)	Negative	Negative	Pregnancy Dental disease
European short hair Female 2 years old	*L. kirschneri* sg Grippotyphosa serovar Grippotyphosa (titre 1:100) *L. interrogans* sg Icterohaemorrhagiae serovar Icterohaemorrhagiae (titre 1:100) *L. interrogans* sg Icterohaemorrhagiae serovar Copenhageni (titre 1:100)	Negative	Negative	Pregnancy
European short hair Male 10 years old	*L. interrogans* sg Canicola serovar Canicola (titre 1:100)	Negative	Negative	Dental disease
European short hair Male 9 years old	*L. interrogans* sg Australis serovar Bratislava (titre 1:200)	Negative	Negative	Abscess (maxillary region) Otitis Conjunctivitis Ectoparasites (Fleas)
European short hair Female 8 years old	*L. kirschneri* sg Grippotyphosa serovar Grippotyphosa (titre 1:100)	Negative	Negative	Dental disease
Passive surveillance				
European short hair Female neutered 4 years old	*L. kirschneri* sg Grippotyphosa serovar Grippotyphosa (titre 1:800) *L. interrogans* sg Icterohaemorrhagiae serovar Icterohaemorrhagiae (titre 1:200) *L. interrogans* sg Icterohaemorrhagiae serovar Copenhageni (titre 1:200)	Negative	Positive	Jaundice Polyuria/polydipsia Acute kidney disease FIV FeLV
European short hair Male neutered 6 years old	*L. interrogans* sg Australis serovar Bratislava (titre 1:1600) *L. interrogans* sg Icterohaemorrhagiae serovar Copenhageni (titre 1:800)	NP (*)	NP (*)	Hyperthermia Haematuria Acute over chronic nephropathy
European short hair Male neutered 6 years old	*L. kirschneri* sg Grippotyphosa serovar Grippotyphosa (titre 1:800)	Negative	Negative	Acute kidney disease Lymphoma
European short hair Male neutered 6 years old	*L. interrogans* sg Australis serovar Bratislava (1:1600)	NP (**)	NP (**)	Dehydration Polyuria Polydipsia Haematuria Feline herpesvirus (FHV-1) Hyperthyroidism Chronic enteropathy

Sexual condition is indicated when information was available. (*): the cat was treated with antibiotics. (**): no urine samples were available for molecular and microbiological assay. NP: not performed.

## Data Availability

The datasets generated during and/or analysed during the current study are available from the corresponding author on reasonable request.
